# *Fasciola hepatica* demonstrates high levels of genetic diversity, a lack of population structure and high gene flow: possible implications for drug resistance

**DOI:** 10.1016/j.ijpara.2016.09.007

**Published:** 2017-01

**Authors:** Nicola J. Beesley, Diana J.L. Williams, Steve Paterson, Jane Hodgkinson

**Affiliations:** a Veterinary Parasitology, Institute of Infection and Global Health, University of Liverpool, Liverpool L3 5RF, UK; b Centre for Genomic Research, Institute of Integrative Biology, University of Liverpool, Liverpool L69 7ZB, UK

**Keywords:** *Fasciola hepatica*, Population genetics, Anthelmintic resistance, Diversity, Self-fertilisation, Gene flow, Microsatellites

## Abstract

*Fasciola hepatica*, the liver fluke, is a trematode parasite of considerable economic importance to the livestock industry and is a re-emerging zoonosis that poses a risk to human health in *F. hepatica*-endemic areas worldwide. Drug resistance is a substantial threat to the current and future control of *F. hepatica*, yet little is known about how the biology of the parasite influences the development and spread of resistance. Given that *F. hepatica* can self-fertilise and therefore inbreed, there is the potential for greater population differentiation and an increased likelihood of recessive alleles, such as drug resistance genes, coming together. This could be compounded by clonal expansion within the snail intermediate host and aggregation of parasites of the same genotype on pasture. Alternatively, widespread movement of animals that typically occurs in the UK could promote high levels of gene flow and prevent population differentiation. We identified clonal parasites with identical multilocus genotypes in 61% of hosts. Despite this, 84% of 1579 adult parasites had unique multilocus genotypes, which supports high levels of genotypic diversity within *F. hepatica* populations. Our analyses indicate a selfing rate no greater than 2%, suggesting that this diversity is in part due to the propensity for *F. hepatica* to cross-fertilise. Finally, although we identified high genetic diversity within a given host, there was little evidence for differentiation between populations from different hosts, indicating a single panmictic population. This implies that, once those emerge, anthelmintic resistance genes have the potential to spread rapidly through liver fluke populations.

## Highlights

• Self-fertilisation does occur but is rare in field populations of *Fasciola hepatica.*
• Some hosts harboured genotypically identical parasites (clones).
• The presence of clones is consistent with clonal expansion and clumped transmission.
• 84% of 1579 *F. hepatica* had unique genotypes, indicating high genetic diversity.
• We found high gene flow, no population structure and low self-fertilisation rate.

## Graphical abstract

**Figure:**
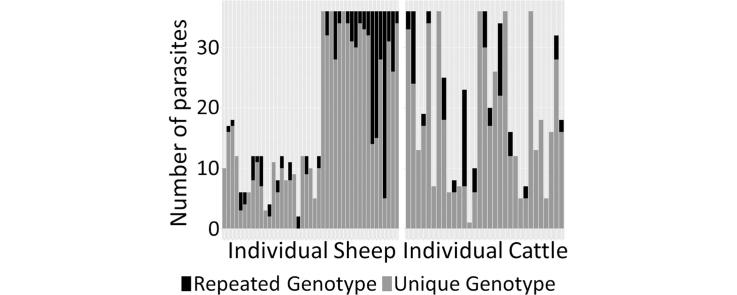

## 1 Introduction

*Fasciola hepatica* is a trematode parasite that causes disease of economic importance in sheep and cattle (Bennett and Ijpelaar, 2005, Schweizer et al., 2005), with an estimated 250 million sheep and 350 million cattle at risk worldwide (Hillyer and Apt, 1997). A zoonosis, it is classed by the World Health Organisation as a neglected tropical disease endemic in human populations in parts of South America, western Europe and Iran (Mas-Coma, 2005, World Health Organisation, 2007, http://www.who.int/neglected_diseases/diseases/en/). Over the last 15–20 years, the diagnosis of *F. hepatica* infection in European livestock has increased (Caminade et al., 2015; https://www.gov.uk/government/uploads/system/uploads/attachment_data/file/458616/vida-cattle-07–14.pdf; https://www.gov.uk/government/uploads/system/uploads/attachment_data/file/458618/vida-sheep-07–14.pdf), possibly due to changing climate, changing farming practices including animal movement and land use, and the emergence of resistance to the drug of choice, triclabendazole (van Dijk et al., 2010, Fairweather, 2011a, Fox et al., 2011, Caminade et al., 2015). Resistance of *F. hepatica* to triclabendazole was first reported in sheep in Australia in 1995 (Overend and Bowen, 1995), and is now frequently reported across Europe and South America (Moll et al., 2000, Gaasenbeek et al., 2001, Álvarez-Sánchez et al., 2006, Mooney et al., 2009, Olaechea et al., 2011, Daniel et al., 2012, Ortiz et al., 2013). It is considered to be a substantial threat to the current and future control of *F. hepatica* (Kelley et al., 2016).

Population genetic analyses are key to understanding the origin, evolution and spread of resistance genes in populations and are thus a vital component of anthelmintic resistance studies (Gilleard and Beech, 2007). They allow us to identify management factors influencing the migration of resistance genes, and so help to mitigate against their spread. It is recognised that the husbandry and management of different farms have the potential to affect the population structure of parasites (Grillo et al., 2007) by influencing the movement of the definitive host and, therefore, *F. hepatica* parasites. Additionally, the age and production system for an animal influences the extent to which it has been exposed to *F. hepatica* on pasture and to what extent it may have been treated with anthelmintics.

A number of aspects of *F. hepatica* biology have the potential to influence genetic diversity and population structure and therefore impact on the spread of genes, including those responsible for anthelmintic resistance (Hodgkinson et al., 2013). Firstly, it is known that clonal expansion of *F. hepatica* occurs within the snail intermediate host, *Galba truncatula* (Thomas, 1883, Krull, 1941). Therefore, there is the potential for multiple metacercariae of the same origin and genotype to exist on pasture, and parasites with the same multilocus genotype (MLG) have been found within, and shared between, definitive hosts (Vilas et al., 2012). Secondly, as a hermaphrodite, *F. hepatica* can self- and cross-fertilise. Self-fertilisation is a form of inbreeding which has the potential to influence allele frequency in a population. If anthelmintic resistance is a recessive trait, a high level of self-fertilisation means there is the potential for resistant alleles to spread more rapidly. Thirdly, clonal expansion in the snail, combined with low levels of infection in the snail population as a whole, could pose a bottleneck to gene flow and lead to population structuring. Finally, *F. hepatica* has a wide host range, infecting multiple species of domestic and wild animals (Parr and Gray, 2000, Vignoles et al., 2001, Vignoles et al., 2004, Arias et al., 2012). This may allow the flow of genes amongst livestock species and maintain a reservoir of genetic diversity in wild animals. In addition, adult *F. hepatica* in the definitive host can be long-lived (Durbin, 1952), and their reproductive capacity may be present for many years in untreated animals.

An understanding of *F. hepatica* genetic diversity has implications for the development and validation of new methods of control. Knowledge of the provenance, infectivity, pathogenicity and resistance status of laboratory isolates is important (Hodgkinson et al., 2013). Laboratory maintained isolates of *F. hepatica* are frequently used in research, including in drug and vaccine trials (Fairweather, 2011b), but are not representative of field isolates. For example, the Cullompton isolate is aspermic and triploid (Fletcher et al., 2004), the Sligo isolate exhibits abnormal spermatogenesis (Hanna et al., 2008), and the Fairhurst isolate is highly homogenous (Walker et al., 2007).

Previously we have shown that the British *F. hepatica* population naturally infecting sheep and cattle is diploid, spermic and predominantly reproduces by sexual reproduction (Beesley et al., 2015). Here, we present the largest population genetic study to date for *F. hepatica*, involving the genotyping of 1579 adult parasites. Adult *F. hepatica* samples were collected from three countries (Scotland, England and Wales) from two definitive host species (sheep and cattle), and MLGs were produced using our panel of microsatellite markers (Cwiklinski et al., 2015a). A proportion of hosts harboured multiple, genotypically identical parasites. However, overall, we found substantial genetic variation within populations infecting a given host and high levels of genetic diversity in the liver fluke population as a whole, but little differentiation between populations infecting sheep and cattle. Our data indicate a lack of geographic or host species structuring in *F. hepatica* from Great Britain and high gene flow, which could promote the emergence and spread of drug resistance in a population. The results of this study may be relevant to other areas where widespread movement of livestock is practised.

## 2 Materials and methods

### 2.1 Populations of *F. hepatica*

Adult *F. hepatica* were recovered from the livers of 44 naturally infected sheep between November 2012 and April 2013, from two abattoirs (Wales and central England, UK). Similarly, parasites were recovered post mortem from 31 cattle livers between October 2013 and January 2014, from an abattoir (Wales, UK). A total of 950 parasites were genotyped from sheep and 629 from cattle (Table 1). The Rapid Analysis and Detection of Animal Related Risks (RADAR), Animal and Plant Health Agency (APHA, UK, https://www.gov.uk/government/organisations/animal-and-plant-health-agency) provided information on the origin of cattle livers. Adult parasites were isolated from the bile ducts and incubated for 2 h at 37 °C in 1–2 ml of DMEM with 120 μg ml^−1^ gentamicin and 120 μg ml^−1^ amphotericin B to allow purging of intestinal contents and eggs. Parasites were snap frozen and stored at −80 °C.

### 2.2 Preparation of DNA template and microsatellite genotyping

A small section of each parasite, anterior to the ventral sucker, to avoid contamination with eggs or sperm, was used for DNA extraction. The tissue was divided into small pieces to ensure efficient lysis. DNA extraction was performed using a DNeasy Blood & Tissue Kit (Qiagen, UK) as per the manufacturer’s instructions and DNA was diluted to 10 ng μl^−1^.

A panel of 15 microsatellites previously validated with 46 adult *F. hepatica* (Cwiklinski et al., 2015a), was applied to each parasite DNA sample to generate an individual MLG. For efficiency the methodology was modified for a multiplex approach; the Type-it Microsatellite PCR kit (Qiagen) was used according to the manufacturer’s instructions (Cwiklinski et al., 2015a). The 15 loci were grouped as follows: (i) Fh_1, Fh_6, Fh_13, Fh_15 annealing temperature 55 °C; (ii) Fh_2, Fh_3, Fh_5, Fh_8, annealing temperature 57 °C; (iii) Fh_9, Fh_10, Fh_11, Fh_14, annealing temperature 57 °C; and (iv) Fh_4, Fh_7 and Fh_12, annealing temperature 59 °C. PCR products were visualised using SYBR Safe DNA stain (Life Technologies, UK) on a 1.5% agarose gel. PCR products were diluted 25-fold in HPLC-grade water (Sigma–Aldrich, UK), and sequenced using an ABI PRISM 3100 Genetic Analyser capillary electrophoresis system (Life Technologies) (Cwiklinski et al., 2015a). Fragment sizes were determined using Peak Scanner v2.0 software (Life Technologies).

### 2.3 Population genetic analyses

Allele frequencies were determined using CERVUS 3.0.7 (Kalinowski et al., 2007; available from www.fieldgenetics.com) and genotype frequencies were determined using GENEPOP 4.2.1 (Rousset, 2008; available from http://kimura.univ-montp2.fr/~rousset/Genepop.htm). Null allele frequency was determined using CERVUS 3.0.7 (Kalinowski et al., 2007). Loci Fh_1, Fh_3, Fh_4, Fh_7, Fh_8 and Fh_14 were identified as having greater than 5% frequency of null alleles (Table 2), therefore these loci, together with locus Fh_9 which produced inconsistent traces, were excluded from the remaining population genetic analyses.

Average heterozygosities were determined for each locus using Arlequin 3.5.1.3 (Excoffier and Lischer, 2010). Unbiased heterozygosity was calculated using GenClone 2.0 (Arnaud-Haond and Belkhir, 2007). Heterozygosity was determined for each individual parasite based on the proportion of loci that were heterozygous. Mann–Whitney *U* tests were performed using Minitab 17. GenClone 2.0 (Arnaud-Haond and Belkhir, 2007) was used to identify repeated MLGs (defined as two or more parasites sharing the same MLG) and to calculate corresponding *P_sex_* values (the probability that a MLG is derived from a distinct reproductive event rather than being from a clonal lineage), which were adjusted using *F*_IS_ values (Parks and Werth, 1993). Animals from the same farms, or that shared repeated MLGs, were grouped when calculating *P_sex_* values. *X*^2^ and Mann–Whitney *U* tests were performed using Minitab 17.

To determine whether repeated MLGs tended to co-occur in the same host (Gregorius, 2005, Criscione et al., 2011, Vilas et al., 2012) a contingency table was created as described by Vilas et al. (2012), and a Fisher’s exact test with a Monte Carlo simulation (5000 replicates) was performed using R 3.0.1 (http://www.R-project.org/). All parasites were analysed together. The presence of repeated MLGs might make alleles appear more common and affect population genetic structure analyses. Therefore, for the remaining analyses repeated MLGs were reduced to one instance.

Deviations from Hardy–Weinberg equilibrium were calculated using GENEPOP 4.2.1 (Rousset, 2008) using a two-tailed exact test with Markov Chain algorithm (10,000 dememorization, 250 batches, 5000 iterations). To determine the extent of any significant deviation from Hardy–Weinberg equilibrium, *F*_IS_ values (Weir and Cockerham, 1984) were calculated using GENEPOP 4.2.1 (Rousset, 2008).

All pairs of loci, with all parasites analysed together, were assessed for linkage disequilibrium using GENEPOP 4.2.1 (Rousset, 2008). Due to the number of tests, *P* values were corrected and compared using (i) Bonferroni correction (*P* < 0.00179 was considered significant) and (ii) false discovery rate correction (*Q* < 0.05 was considered significant) (Benjamini and Hochberg, 1995), the latter performed using R 3.0.1 (http://www.R-project.org/). To demonstrate the extent of linkage disequilibrium for any pair of loci with significant *P* values, r^2^ values were calculated. To calculate this value, knowledge of the gametic phase is needed. Since this is unknown here, the ELB algorithm (Excoffier et al., 2003) was used to infer the gametic phase. These calculations were performed using Arlequin 3.5.1.3 (Excoffier and Lischer, 2010).

Genotypic richness (Dorken and Eckert, 2001) was used to describe genetic diversity, calculated using GenClone 2.0 (Arnaud-Haond and Belkhir, 2007). When calculating genotypic richness, animals from the same farms, or that shared the same MLG, were grouped. Mann–Whitney *U* tests were performed using Minitab 17.

*F*_IS_ and *F*_ST_ values were calculated using GENEPOP 4.2.1 (Rousset, 2008), and confidence intervals (CIs) were calculated using FSTAT 2.9.3 (Goudet, 1995; available from http://www2.unil.ch/popgen/softwares/fstat.htm). The rate of self-fertilisation (s) was calculated from the *F*_IS_ values using the equation *F*_IS_ = s/(2 − s). Pairwise *F*_ST_ values were calculated using Arlequin 3.5.1.3 (Excoffier and Lischer, 2010). Principal component analysis (PCA) of these values was performed in R 3.0.1 (http://www.R-project.org/), and the package ggplot2 was used to plot results. GENEPOP 4.2.1 (Rousset, 2008) was used to produce a measure for the average number of migrants between populations (*N*_m_) using the private allele method developed by Slatkin (1985). For this calculation, parasites were grouped according to the definitive host from which they originated.

Isolation by distance testing was possible for parasites from cattle only, as farm location was known. Parasites were grouped into populations dependent upon farm of origin. Isolation by distance was then tested using GENEPOP 4.2.1 (Rousset, 2008). A Mantel test (5000 permutations) was performed using log transformed geographic distances with the minimum geographic distance set at 0.0001. Data were plotted in R 3.0.1 (http://www.R-project.org/) using the package ggplot2.

Structure 2.3.4 (Pritchard et al., 2000; available from http://pritchardlab.stanford.edu/structure.html) was used to detect population structure. To determine the ancestry of individuals, the admixture model with default settings was chosen. This allows for an individual to have mixed ancestry. For the allele frequency model, allele frequencies were correlated amongst populations with default settings. Burn-in length was set at 200,000 and was followed by 100,000 Markov Chain Monte Carlo repeats. *K* was set at 1–47 (the number of farms animals came from) and repeated 20 times. To determine the most appropriate value for *K*, Δ*K* was determined using the method proposed by Evanno et al. (2005) and calculated using Structure Harvester (Earl and vonHoldt, 2012; available from http://taylor0.biology.ucla.edu/structureHarvester/). Data were plotted in R 3.0.1 (http://www.R-project.org/) using the packages ggplot2 and gridExtra. Unless otherwise stated *P* < 0.05 was considered significant.

### 2.4 Ethical approval

Ethical approval was received from the University of Liverpool’s Veterinary Research Ethics Committee, UK (VREC106 and VREC145).

## 3 Results

### 3.1 Microsatellite genotyping using a multiplex approach

Summary statistics are shown for the microsatellite panel in Table 2. Eight loci (Fh_2, Fh_5, Fh_6, Fh_10, Fh_11, Fh_12, Fh_13 and Fh_15) were used to produce a MLG for all 1579 parasites. Only locus Fh_2 showed significant deviation from Hardy–Weinberg equilibrium, however the *F*_IS_ value at this locus was low so the deviation was considered minor (Table 2). Each pair of loci was assessed for evidence of linkage disequilibrium. Five pairs of loci showed significant *P* values (*Q* < 0.005 using false discovery rate; *P* < 0.00179 using Bonferroni correction) but low *r*^2^ values (median = 0.0001, range 0–0.33), indicating that the pairs of loci are closer to equilibrium than disequilibrium.

### 3.2 Genetically identical (clonal) parasites are common in British *F. hepatica* infections

**Fig. 1:**
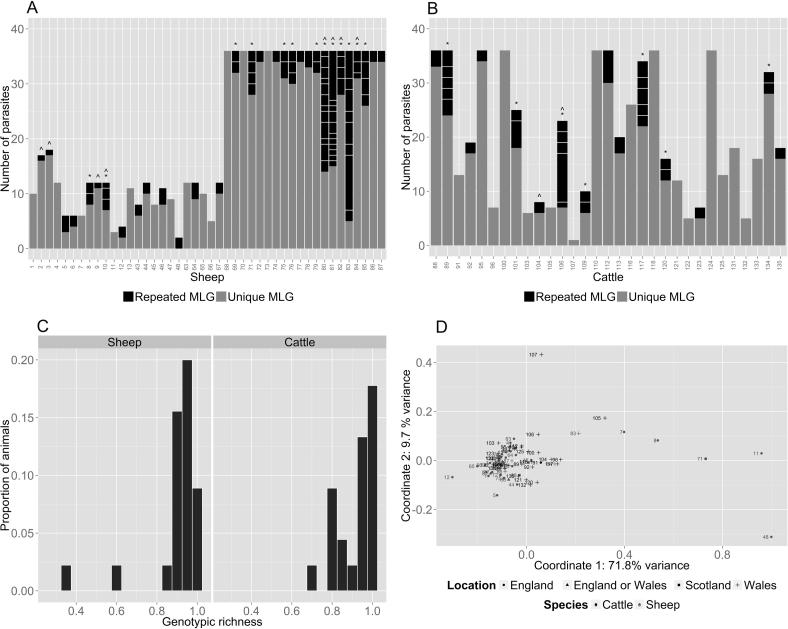
Representation of the number of clonal *Fasciola hepatica* parasites (those with repeated multilocus genotypes) found within each individual (A) sheep and (B) cattle and shown as a proportion of the total number of parasites genotyped from each definitive host; numbers on the x-axis are individual animal identifiers; ^∗^ indicates that more than one clone set was found in an individual host, the bar is split to distinguish the number of parasites within each clone set; ^^^ indicates that clone sets are shared between hosts. (C) Histogram displaying the genotypic richness values within each definitive host, separated into sheep and cattle. Genotypic richness (*R*) is a measure of genetic diversity and is calculated as *R* = (*G* − 1)/(*N* − 1) where *G* = the number of genotypes identified in each host and *N* = the number of parasites genotyped; each histogram bar is of width 0.05 with the bar centred over the upper limit. (D) Principal Component Analysis for pairwise *F*_ST_ values between the parasites of each definitive host. Each data point and its corresponding number represent an individual animal, and the shape and colour of the symbol represent the location and species of that animal, respectively.

Given that the life cycle of *Fasciola* spp. involves clonal expansion within the snail host, and release of genetically identical cercariae onto pasture, we tested whether multiple parasites within a liver exhibited the same MLG. Overall, 71% of sheep and 48% of cattle livers harboured clonal parasites (this difference was not statistically significant, *X*^2^ = 0.588; *P* = 0.4432). A total of 96 parasite genotypes were represented more than once, with the majority, 65 genotypes, shared by just two parasites. Sixteen of the animals showed evidence of infection with more than two parasites of the same genotype, with a maximum of 10 clonal parasites reported in one sheep. Fig.1A, B show the number of unique and repeated MLGs (defined as an MLG present more than once) within each individual definitive host. There were a number of animals where multiple different MLGs were shared by parasites, with a maximum of eight distinct MLGs observed in a single animal. This happened on two occasions, sheep 80 and sheep 83 (Fig.1A).

Generally, parasites with the same MLG were present within the same animal, and it was found that repeated MLGs did tend to co-occur in the same host (Fisher’s exact test with Monte Carlo simulation *P* = 0.0002). However, repeated MLGs were also found to be shared between individual sheep (sheep 2 and 3; sheep 9 and 10; sheep 80 and 81; sheep 82 and 84) and cattle (cattle 104 and 106), but clonal parasites were not found to be shared by both sheep and cattle. In total, 16% of all parasites identified in sheep and cattle lacked a unique MLG and the proportion was significantly higher in sheep than cattle (*X*^2^ = 4.9052; *P* = 0.02678). However, this was not because parasite burdens in sheep were higher, since burdens for sheep and cattle were not significantly different (Mann–Whitney *U* test *P* = 0.5842). In order to determine whether those MLGs that occurred more than once in an animal represented different reproductive events or were from the same clonal lineage, *P_sex_* values were calculated. All the *P_sex_* values were highly significant at *n* = 2 and overall ranged from 1.74 × 10^−71^ to 3.4 × 10^−4^ in parasites from sheep and from 2.97 × 10^−47^ to 2.39 × 10^−5^ in parasites from cattle. This supports the conclusion that the repeated MLGs represent parasites arising from clonal lineages.

### 3.3 *Fasciola hepatica* in Great Britain is genetically diverse

Inbreeding and clonal expansion in *F. hepatica* may impact levels of genetic diversity in *F. hepatica* populations, hence we genotyped a large number of parasites from multiple sheep and cattle throughout Great Britain. The heterozygosity of individual parasites, a measure of genetic variation, ranged from 0.25 to 1, whilst the mean heterozygosity of all parasites across all loci was 0.752 (SD = 0.130), suggesting high levels of genetic variation in the overall population. In the majority of cases, 29 animals, each parasite genotyped had a unique MLG (Fig.1A, B). Genotypic richness (*R*), the measure of genetic diversity that describes the number of distinct MLGs within a population, was high, *R* = 0.901. As with heterozygosity a range of values for *R* were reported within individual definitive hosts, 0.343–1.0, however, parasites in the majority of animals showed a genotypic richness >0.8 (Fig.1C). These analyses confirmed that the British *F. hepatica* population demonstrated high genetic diversity.

### 3.4 *Fasciola hepatica* from sheep and cattle are not genetically distinct

Given that both sheep and cattle can be infected with *F. hepatica* and often co-graze, we asked whether there is evidence of population structuring between the two hosts. The pairwise *F*_ST_ between parasites from sheep and cattle was 0.00145. Although this value was statistically significant (*P* < 0.05) given the large sample size, a value of less than 1% indicates little genetic differentiation between parasites from sheep and cattle. Furthermore, PCA of pairwise *F*_ST_ values between the parasites within each definitive host does not reveal any clustering based on host species (Fig.1D). No significant difference in the level of genetic variation and diversity was seen when parasites from sheep and cattle were assessed separately: heterozygosity across all loci was 0.758 (SD: 0.141) in sheep and 0.745 (SD: 0.118) in cattle (Mann–Whitney *U* test *P* *=* 0.092), and the genotypic richness across all parasites was 0.890 in sheep and 0.918 in cattle (Mann–Whitney *U* test *P* = 0.689). Sheep and cattle share a number of common alleles and genotypes (Table 3) but private (unique) alleles were also identified for each host species, with 14.7% and 6.0% of all alleles unique to sheep and cattle, respectively. The most common allele at each locus was identical for both host species, with the exception of loci Fh_2 and Fh_4 (Table 3; data not available for locus Fh_1). The most common genotypes were also identical at nine loci (Fh_5, Fh_7, Fh_8, Fh_9, Fh_10, Fh_11, Fh_12, Fh_14 and Fh_15; Table 3). Therefore, parasites from sheep and cattle showed not only a similar level of genetic variation, but also largely similar alleles and genotypes. From the evidence presented in this study there does not appear to be structuring of the parasites from sheep and cattle, and *F. hepatica* infecting the two species of definitive host are genetically similar.

### 3.5 High gene flow exists in *F. hepatica* populations from Great Britain

**Fig. 2:**
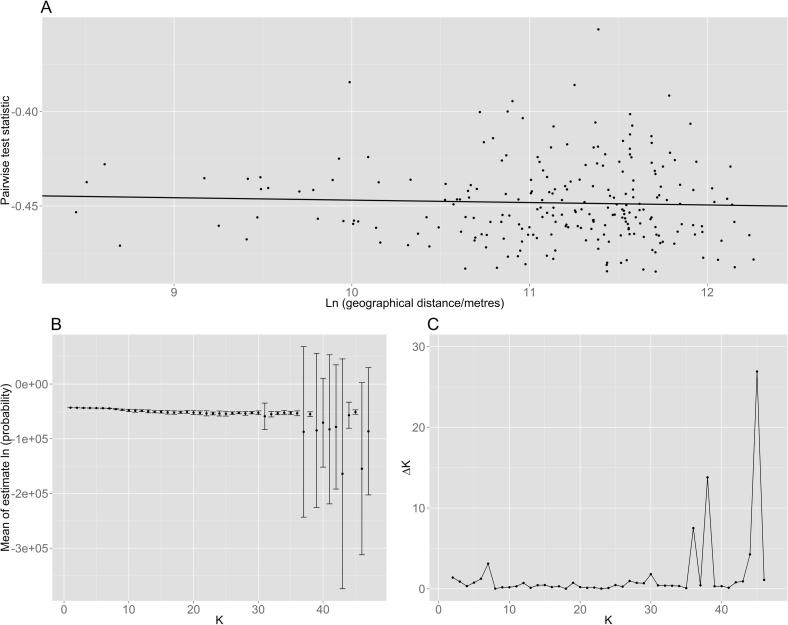
Results of tests for isolation by distance and population structuring within *Fasciola hepatica* from Great Britain. (A) Isolation by distance results for *F. hepatica* parasites from cattle. Each point plots the genetic difference (pairwise test statistic based on *F*_ST_/[1 − *F*_ST_]) against the geographical distance (on a natural logarithm (Ln) scale) between each pair of populations. Each population consists of the parasites on one farm; comparisons are not made between parasites on the same farm. The regression line is shown and has the following parameters: slope = −0.00129 (95% confidence interval = −0.00317, 0.00142); intercept = −0.434; *P* = 0.2968. Therefore, there is no evidence of isolation by distance as the slope is negative and the *P* value non-significant. (B) Structure (Pritchard et al., 2000) was used to detect population structure. *K* represents the number of populations assumed for each simulation and is plotted against the mean natural log probabilities. Each simulation was repeated 20 times and error bars show the SDs. (C) To determine the most appropriate value for *K*, Δ*K* (the rate of change in the log probability between successive K values; Evanno et al., 2005) was determined using Structure Harvester (Earl and vonHoldt, 2012). The results indicate a single population with no structure.

The extent of gene flow amongst *F. hepatica* populations was investigated, given that widespread movement of sheep and cattle is commonly practiced in the UK. The evidence from a number of our analyses indicates that in Great Britain, *F. hepatica* represents a single panmictic population with no geographic structuring. PCA of pairwise *F*_ST_ from locations up to 650 km apart showed there was no clustering based on the location of the definitive host (Fig.1D). Similarly, there was no evidence of isolation by distance (exact location information was available for cattle only) since the slope of the regression line was negative, and the *P* value was non-significant (Fig.2A). The mean likelihood results from Structure (Pritchard et al., 2000) did not reach an asymptote which would be expected if the population was structured (Fig.2B). In addition the majority of Δ*K* values were low (Fig.2C), indicating a single population with no structure. Finally, *F*_ST_ analysis between definitive hosts (across all parasites and loci) was 0.0202, which was low, supporting little genetic differentiation and low levels of population structure. This lack of genetic differentiation infers high gene flow in the population. When parasites from sheep and cattle were assessed separately, the *F*_ST_ values amongst sheep and amongst cattle were very similar: 0.0193 and 0.0207, respectively. Since private alleles were identified, *N*_m_ (the effective number of migrants) can be used to give an indirect estimate of gene flow. Parasites were grouped based on the definitive host from which they were collected, giving a mean sample size of 18.99. *N*_m_ across all loci was 5.59, and since this means the number of migrants per generation into the population is greater than 2, it is indicative of high gene flow (Slatkin, 1985). Similarly, when parasites from sheep and cattle were assessed separately, *N*_m_ values were 6.85 and 8.20, respectively. Therefore both the *F*_ST_ and *N*_m_ values support a high level of gene flow in the UK *F. hepatica* population.

### 3.6 Low levels of self-fertilisation occur in *F. hepatica* populations from Great Britain

Self-fertilisation will result in loss of genetic diversity within individual parasites, which can be estimated from Wright’s *F*_IS_ statistic. *F*_IS_ across all loci and parasites was 0.0011, which was not significantly different from zero (95% CI: −0.011, 0.013), and indicated a selfing rate no higher than 2%.

## 4 Discussion

This study has provided valuable insights into aspects of *F. hepatica* population biology. The fact that the selfing rate was estimated to be no greater than 2% suggests that self-fertilisation can occur but it is rare in the field. Clonal parasites with identical MLGs were identified in 61% of definitive hosts, implying that clones are commonly found in *F. hepatica* infections, a finding that is consistent with earlier studies (17 of 20 animals; Vilas et al., 2012). We found parasites with identical MLGs were usually in the same host (Fig.1A, B) and when clonal parasites were found to be shared between animals, each pair of animals was from the same geographic area and typically from the same farm. Our findings indicate that, following clonal expansion in the snail, there is aggregation of infective clonal metacercariae on pasture, with little mixing of parasites prior to ingestion by the definitive host. The life cycle of *F. hepatica* lends itself to clumped transmission in several ways. Firstly, a single miracidium infecting a snail produces multiple (e.g. mean 114.9; SD 80.3; Dreyfuss et al., 1999) genetically identical cercariae. Secondly, snails are known to shed multiple cercariae at the same time (Hodasi, 1972, Dreyfuss et al., 2006). Thirdly, reported levels of *F. hepatica* infection in *G. truncatula* in the UK and the Republic of Ireland can be as low as 3% (Crossland et al., 1969, Relf et al., 2011). Finally, snail habitats tend to be small (Rondelaud et al., 2011), which may concentrate metacercariae in small areas of pasture. However, it is important to appreciate that mortality can occur at every stage of the life cycle (Ollerenshaw, 1959), thus potentially limiting the survival of clonal parasites. Indeed, the maximum number of clonal adult parasites in any one host was 10 out of the 36 parasites genotyped (Fig.1A). The fact that *P_sex_* values were significant indicated that parasites with identical MLGs arose from the same clonal lineage rather than distinct reproductive events, which would be consistent with the findings of Vilas et al. (2012). Neither our study nor Vilas et al. (2012) reported parasites with the same MLG in both sheep and cattle. Whilst it would be expected that sheep and cattle that were known to co-graze might be more likely to be infected with the same clonal lineage, parasites with the same composite mitochondrial haplotypes have been reported in sheep and cattle from distinct counties of Northern Ireland (Walker et al., 2007).

Despite the presence of clonal parasites in sheep and cattle, these constituted only 16% of the total parasite population under study as the majority of the 1579 parasites analysed had unique MLGs. Our analysis of the population as a whole indicated that the British *F. hepatica* population was highly genetically diverse (Fig.1C). Undoubtedly, one of the best ways to maintain this diversity is the capacity for *F. hepatica* to reproduce in the definitive host through meiosis, and the relative proportion of self- and cross-fertilisation, as well as the genetic implications of parthenogenesis, are important considerations (Hanna et al., 2016). Our findings on low selfing rates indicate that cross-fertilisation predominates in *F. hepatica*. Recently, it has been observed that parasites with higher heterozygosity levels were more likely to establish in the liver following infection (Zintl et al., 2015), raising the possibility that host selection enhances the likelihood of cross-fertilisation.

Of particular interest here is the fact that we sampled lambs that had grazed for only one season, yet they displayed highly diverse adult parasite populations, equivalent to those seen in cattle that had grazed over several seasons; a point which has been alluded to previously by Walker et al. (2007). This suggests that the metacercariae on pasture to which the lambs were exposed were also highly genetically diverse. Clonal expansion and low levels of infection in snails present a potential genetic bottleneck and raise the question of how *F. hepatica* maintains its genetic diversity. It is known that, experimentally, snails can be infected with two miracidia 4 h apart (Dreyfuss et al., 2000, Dar et al., 2011) and, in the field, snails have been found to be infected by more than one miracidium (Rondelaud et al., 2004). If a snail can be simultaneously infected with multiple miracidia and subsequently shed cercariae of many genotypes, this could drive genetic diversity. Snail habitats can be difficult to locate and whilst the level of infection within snails has been reported to be as low as 0.8% (Rondelaud and Dreyfuss, 1997), it is possible that levels of infection in the snail are considerably higher. There is also evidence that *F. hepatica* can infect snails other than *G. truncatula* (Abrous et al., 1999, Rondelaud et al., 2001, Dreyfuss et al., 2005, Relf et al., 2009, Caron et al., 2014). Furthermore, given that snails infected with *F. hepatica* have been found in areas with no ruminant contact (Dreyfuss et al., 2003), wild definitive hosts such as rabbits and deer could function as important reservoir hosts in maintaining diversity (Parr and Gray, 2000, Arias et al., 2012). Another possible way to maintain genotypic diversity is via the long-term survival of metacercariae on pasture. Metacercariae have been reported to be both viable and infective for at least 130 days at 10 °C (Boray, 1969), but we have no knowledge of how long metacercariae survive in the field, yet this has important implications for control. At a practical level given that efficacy of drugs and vaccines can be compromised by the presence of genetic diversity, an important understanding of this standing genetic variation is essential to the rational selection of new vaccine candidates/drug targets for *F. hepatica*.

There is the potential for husbandry and management practices to affect the population structure of parasites (Grillo et al., 2007). Our analysis of the British *F. hepatica* population showed no evidence of structuring geographically or amongst parasites from sheep and cattle (Figs. 1D, 2B, C), indicating panmixia and high gene flow. It has been suggested that movement of the definitive host is a key factor in maintaining high levels of gene flow in *F. hepatica* (Semyenova et al., 2006, Bazsalovicsová et al., 2015). Livestock in the UK are frequently moved around and between countries, and it is likely that the movement of livestock in Great Britain contributes to the high gene flow observed. Even a small amount of migration can destroy any observed population structure, giving the appearance of panmixia (Wright, 1931); for example moving animals to a new farm could introduce a new population of parasites as well as exposing the definitive host to a different resident parasite population. Whilst further analysis of parasites from flocks or herds where animal movement is restricted, or ideally ‘closed’, may reveal structure not previously detected, panmixia is not merely a feature of British *F. hepatica* populations; similar findings have been reported in Spain and Bolivia (Hurtrez-Boussès et al., 2004, Vázquez-Prieto et al., 2011). The results of this study may be relevant to other areas where widespread movement or importation of livestock is practised. In support of this, identical mitochondrial haplotypes found between flukes isolated from the Republic of Ireland and Greece was attributed to importation of animals (Walker et al., 2007). It would be interesting to determine the level of genetic diversity in, and genetic differentiation between, populations of *F. hepatica* from wild, as opposed to farmed, definitive hosts.

Resistance to triclabendazole has been reported widely throughout the UK (Daniel et al., 2012, Gordon et al., 2012, Hanna et al., 2015). Investigation of triclabendazole resistance in fluke in laboratories worldwide has resulted in the pursuit of a number of potential candidate genes and biological pathways (reviewed by Kelley et al., 2016). The precise loci and, therefore, genes involved are still to be defined but a genome-wide approach is currently underway to identify the major genetic determinant of triclabendazole resistance (Hodgkinson et al., 2013). Our findings have implications for the emergence and spread of anthelmintic resistance. In terms of emergence, we have shown that there is high standing genetic variation in British *F. hepatica* populations, which may include rare genetic variants able to confer resistance to anthelmintics (Gilleard, 2013). This is consistent with the observation of high levels of coding variation reported within the *F. hepatica* genome for UK isolates (Cwiklinski et al., 2015b). While the treatment history, and thus triclabendazole resistance status, of the parasites analysed here was not known, high mitochondrial diversity has been reported in wild type parasites that survived treatment with triclabendazole, as well as the triclabendazole-resistant Oberon laboratory isolate (Walker et al., 2007). Although we have shown that self-fertilisation is not the norm in *F. hepatica* populations from Great Britain, any adult fluke with a resistant genotype that remains following drug treatment would be able to exploit this aspect of their biology to reproduce and contaminate the pasture. Thereafter, our results indicate that clonal expansion within the snail intermediate host, coupled with clumped transmission, could act to propagate these resistant genotypes within a farm and increase the likelihood of resistant genotypes mating within a host. In relation to the spread of resistance, in the UK sheep are treated with anthelmintics against *F. hepatica* more often than cattle and resistance to triclabendazole is more frequently reported in parasites infecting sheep (Sargison et al., 2010). However, our findings indicate that drug-resistant *F. hepatica* from sheep could be readily transferred to cattle. Furthermore, since there is no evidence of structuring either geographically or between parasites from sheep and cattle, this means anthelmintic resistance has the potential to spread around the country, compounded by the movement of animals and maintained in wildlife reservoirs.

We have used microsatellite markers to show that *F. hepatica* populations in the field are genetically diverse and outbred. Thus, despite the ability of *F. hepatica* to self-fertilise within the definitive host and to clonally multiply within the intermediate host, there is little difference between the genetic structure of *F. hepatica* and that of any other sexually reproducing parasite. The fact that some hosts were infected with parasites of identical MLG indicates clumped transmission to the definitive host, which may be due to aggregation of infective stages on pasture. Adult *F. hepatica* isolated from naturally infected sheep and cattle in Great Britain were found to be highly genetically diverse within the definitive host, but there was little genetic differentiation between populations. This level of genetic diversity is not a product of grazing over time, since the genetic diversity of adult parasites infecting lambs grazing for only one season was similar to that of cattle grazing over several seasons. The genetic diversity reported here implies drug resistance loci will be recombining freely within the genome. Coupled with the high gene flow exhibited by *F. hepatica* populations, this has implications for the emergence and spread of anthelmintic resistance in *F. hepatica* populations.

## Figures and Tables

**Table 1:** *Fasciola hepatica* populations collected from sheep and cattle in Great Britain.

Species	No. of animals	Demographic information		Median burden (range)	No. of parasites genotyped (median; range per liver)
Sheep^a^	8	Scotland		69 (36–>200)	288^b^
Sheep^a^	5	Wales			180^b^
Sheep^a^	1	England			36^b^
Sheep^a^	6	England or Wales			216^b^
Sheep^a^	24	Five farms local to the abattoir in Wales or Central England		9.5^c^ (3–100)	230 (10.5; 2–18)
Cattle	1	England^d^	Males and females, beef and dairy breeds, median age 8.5 years (range 2.0–16.6)^d^	19 (1 –>230)	13
Cattle	30	21 farms in Wales^d^			616 (18; 1–36)

a Samples from lambs (approximately 6–12 months old) that were exposed to *F. hepatica* metacercariae over a period of 3–9 months in the summer and autumn 2012.

b 36 parasites were sampled from each animal.

c Total enumeration was not performed for six animals.

d This information was provided through Rapid Analysis and Detection of Animal-related Risks (RADAR), Animal and Plant Health Agency (APHA, UK, https://www.gov.uk/government/organisations/animal-and-plant-health-agency

**Table 2:** Summary statistics for the microsatellite panel based on 1579 *Fasciola hepatica* from sheep and cattle in Great Britain.

Locus	Frequency of null alleles^a^	No. of alleles exhibited	No. of genotypes exhibited	*H_obs_/H_nb_*	*F*_IS_^b^
Fh_1	**0.5922**^c^	9^c^	17^c^	ND	ND
Fh_2	0.0112	28	109	0.823/0.843	*0.0299*^d^^,^^e^
Fh_3	**0.1252**	7	17	ND	ND
Fh_4	**0.0753**	16	83	ND	ND
Fh_5	0.0097	39	177	0.852/0.867	0.0199
Fh_6	0.0098	30	178	0.885/0.903	0.0082
Fh_7	**0.1051**	11	37	ND	ND
Fh_8	**0.2255**	16	55	ND	ND
Fh_9	−0.1378	2	3	ND	ND
Fh_10	0.0160	17	75	0.797/0.823	0.0327
Fh_11	0.0237	15	68	0.802/0.840	0.0442
Fh_12	0.0051	15	66	0.733/0.740	0.0061
Fh_13	−0.0058	12	28	0.633/0.628	0.0006
Fh_14	**0.2794**	18	75	ND	ND
Fh_15	0.0064	10	21	0.494/0.505	0.0198

*H_obs_*, observed heterozygosity; *H_nb_*, unbiased heterozygosity; MLGs, multilocus genotypes; ND, not determined.

a Allele frequencies were calculated using CERVUS 3.0.7 (Kalinowski et al., 2007) with results in bold indicating greater than 5% null allele frequency.

b *F*_IS_ values are given to indicate deviations from Hardy–Weinberg equilibrium with those results in italics indicating significant *P* values when using the two-tailed exact test – Bonferroni and false discovery rate corrections were applied.

c Values for locus Fh_1 were determined for 720 of the parasites from sheep only.

d Values significant when Bonferroni correction applied (*P* = 0.00625).

e Values significant when false discovery rate correction applied.

**Table 3:** Frequency and identity of the most common alleles and genotypes at each locus for *Fasciola hepatica* isolated from sheep and cattle in Great Britain.

Locus	Most common allele^a^ (frequency)			Most common genotype^a^ (frequency)		
	Parasites from sheep	Parasites from cattle	Parasites from sheep and cattle	Parasites from sheep	Parasites from cattle	Parasites from sheep and cattle
Fh_1	10 (0.32)	ND	ND	1010 (0.26)	ND	ND
Fh_2	08 (0.23)	17 (0.24)	08 (0.22)	0818 (0.098)	0817 (0.11)	0817 (0.095)
Fh_3	08 (0.50)	08 (0.47)	08 (0.49)	0708 (0.35)	0808 (0.29)	0708 (0.32)
Fh_4	19 (0.19)	17 (0.22)	17 (0.20)	1819 (0.080)	1717 (0.086)	1819 (0.073)
Fh_5	27 (0.23)	27 (0.20)	27 (0.22)	2427 (0.083)	2427 (0.085)	2427 (0.084)
Fh_6	15 (0.21)	15 (0.20)	15 (0.21)	1530 (0.056)	1515 (0.048)	1530 (0.049)
Fh_7	13 (0.41)	13 (0.44)	13 (0.42)	1313 (0.22)	1313 (0.24)	1313 (0.23)
Fh_8	12 (0.29)	12 (0.32)	12 (0.30)	1212 (0.16)	1212 (0.18)	1212 (0.17)
Fh_9	07 (0.62)	07 (0.64)	07 (0.63)	0607 (0.65)	0607 (0.56)	0607 (0.62)
Fh_10	09 (0.35)	09 (0.33)	09 (0.34)	0909 (0.12)	0909 (0.14)	0909 (0.13)
Fh_11	13 (0.28)	13 (0.32)	13 (0.30)	1313 (0.096)	1313 (0.13)	1313 (0.11)
Fh_12	10 (0.43)	10 (0.48)	10 (0.45)	1010 (0.19)	1010 (0.25)	1010 (0.21)
Fh_13	08 (0.55)	08 (0.50)	08 (0.53)	0808 (0.31)	0815 (0.31)	0808 (0.28) and 0815 (0.28)
Fh_14	17 (0.24)	17 (0.27)	17 (0.25)	1717 (0.14)	1717 (0.15)	1717 (0.15)
Fh_15	14 (0.64)	14 (0.64)	14 (0.64)	1414 (0.41)	1414 (0.41)	1414 (0.41)

ND, not determined.

a Alleles are identified by the number of repeats and are in a two-figure format (e.g. 08 indicates the most common allele has eight repeats of the microsatellite), with genotypes in a four-figure format made up of two alleles (e.g. 0818 indicates the most common genotype is made up of the alleles 08 and 18 having eight and 18 repeats of the microsatellite, respectively).
